# Primary bone lymphoma of the left radius: a case report and related literature review

**DOI:** 10.1186/2047-783X-19-19

**Published:** 2014-04-09

**Authors:** Min Liu, Bailong Liu, Fujun Han, Yanqiu Song

**Affiliations:** 1 Department of Radiation Oncology, The First Hospital, Jilin University, 71 Xinmin Street, Changchun 130021, China; 2 Cancer Center, The First Hospital, Jilin University, 71 Xinmin Street, Changchun 130021, China

**Keywords:** Bone lymphoma, Chemotherapy, Radiotherapy

## Abstract

Primary bone lymphoma (PBL) is a rare but distinct clinicopathological disease. Because it is not common, the optimal treatment strategy has not been established. Here, we present a patient with PBL of the left radius and review the related literature. We focus on the standard treatment for PBL. Many aspects such as rehabilitation, local control and overall survival need to be considered. Studies on this disease should be carried out to clarify the optimal treatment in the future.

## Background

Primary bone lymphoma (PBL) is a distinct disease. Here, we present a patient with PBL who received combined therapy with radiotherapy, chemotherapy, surgery and rituximab. Additionally, related reports of PBL are reviewed. Further studies are needed to establish the standard strategy for PBL.

## Case presentation

**Figure 1:**
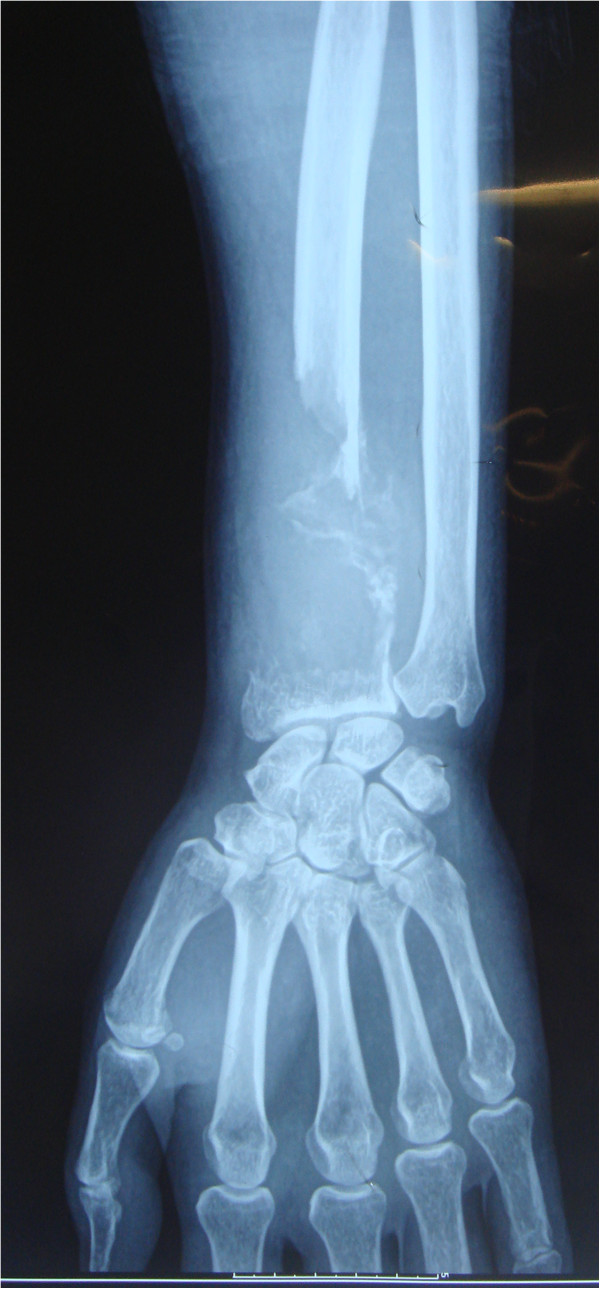
X-ray radiograph showed a large osteolytic lesion of the distal region of the left radius.

**Figure 2:**
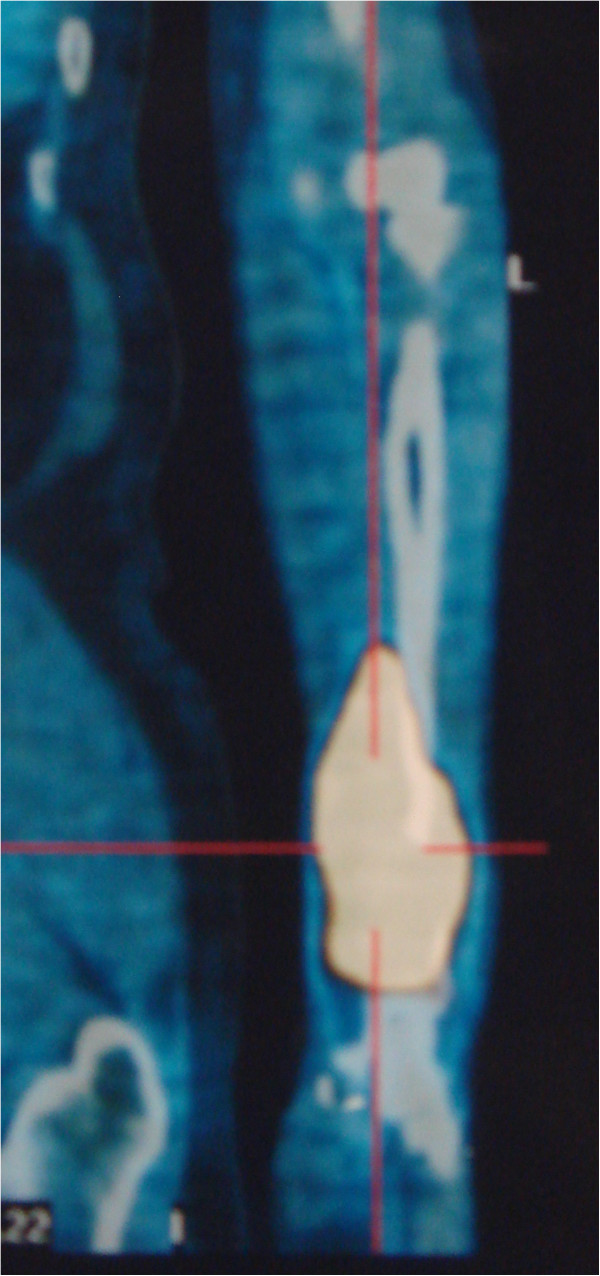
**Pre-therapeutic PET/CT showed intensive FDG uptake in the distal region of the left radius.** FDG, fluorodeoxyglucose; PET/CT, positron emission tomography-computed tomography.

A 53-year-old man presented at the First Hospital of Jilin University (Changchun, China) in December 2011 with a history of progressive pain of the left wrist for about 2 months. The X-ray radiograph of the left upper extremity showed a large osteolytic lesion of the distal radius (Figure 1). The bone biopsy from another hospital revealed B cell non-Hodgkin lymphoma. The immunochemistry showed: LCA(+), CD20(+), BCL-2(slightly +), CD79a(-), CD3(-), CD43(-), CD56(-), CD138(-), CD38(-), κ(-), λ(-) and Ki-67(70%+). The patient then went to the tertiary hospital for consultation of the pathologic results. The definitive diagnosis was diffuse large B cell lymphoma, derived from active B cells out of the germinal center. The immunochemistry revealed: CD10(-), BCL6(+), MUM-1(+), CD5(-) and cyclin D1(-). Positron emission tomography-computed tomography (PET/CT) suggested that there was extremely intense focal uptake in the distal left radius and no focal uptake in other sites (Figure 2). The patient did not have fever, night sweats, pruritus or weight loss.

Laboratory investigations demonstrated a normal complete blood cell count and a generally normal serum biochemical profile. β_2_ microglobulin was 2.30 mg/L. Erythrocyte sedimentation rate was 17 mm/h. Lactate dehydrogenase was normal. The bone marrow aspiration did not find neoplastic involvement. Thus, a primary bone lymphoma, Ann Arbor stage I, International Prognostic Index score 0, was diagnosed.

First, the patient received radiotherapy. When the dose reached 28 Gy/14 f, we gave a cycle of concurrent chemotherapy of rituximab, cyclophosphamide, vincristine and prednisone (R-COP), and the fraction dose was reduced to 1.5 Gy. The total dose was 28 Gy/14 f plus 12 Gy/8 f. The patient then received a cycle of single rituximab. On 1 February 2012, surgery was performed to remove the tumor at the distal region of the left radius, and autograft of the fibula was done and then internally fixed. Thirty radioactive iodines were implanted into the primary tumor bed during the surgery. The post-operative PET-CT did not find any intensive focal uptake. The patient then received a cycle of single rituximab, rituximab, cyclophosphamide, doxorubicin, vincristine and prednisone (R-CHOP) and CHOP, respectively. To date, the patient’s condition remains stable and disease-free survival (DFS) has reached nearly 2 years.

## Discussion

PBL is a rare but distinct clinicopathological entity. It accounts for approximately 5% of all non-Hodgkin lymphomas and 3% of all primary bone tumors [1]. Because it is not common, many aspects of the diagnosis, management and prognosis of this tumor remain controversial.

PBL occurs in a wide spectrum of patients, aged from 1 year and 6 months to 86 years [2], commonly between 20 and 50 years of age, and is more common in males. Diffuse large B cell lymphoma is the most common pathological type, accounting for about 80% of PBLs [3-7]. The most frequent sites of involvement in PBL are the vertebra, pelvis, femur, humerus, skull and rib [3]. Common clinical manifestations include local pain and swelling [1].

Ford *et al*. showed that combined modality treatment (CMT) provided both disease control and survival benefits for PBL compared with single treatment [8]. However, the role of radiotherapy for advanced PBL was challenged in a report by Ramadan *et al*. [3]. The addition of radiotherapy to chemotherapy for advanced cases reduced the 10-year overall survival from 56% to 25%. Whether CMT should be the standard option for PBL requires more research. Achemlal *et al*. reported a case in which rituximab resulted in remarkable improvement for a chemo-resistant PBL [9]. Ramadan *et al*. insisted that irradiation should be carried out when spinal cord compression or residual tumor after chemotherapy existed, which seemed that irradiation only played a minor role [3]. On the contrary, a prospective trial emphasized the importance of radiotherapy to improve the local control and recommended a higher dose (40 to 45 Gy) than that for other types of lymphoma [10]. A retrospective, multicenter Rare Cancer Network (RCN) study also presented a good outcome offered by chemoradiotherapy for early stage of PBL [11]. For a case of PBL of the left mandible, Bosch-Barrera *et al*. reported that chemotherapy (R-CHOP) plus consolidate radiotherapy can result in tumor-free survival of 28 months [12].

Orthopedic intervention is usually needed for pathologic fractures, avascular necrosis, spinal cord compression, or for lesions of the weight-bearing bones compromising stability or joint motion. The potential for long-term survival suggests the use of implants and techniques that have the best chance of long-term control [13]. In our case, concurrent radiotherapy and chemotherapy were given to rapidly eliminate the tumor. However, pathological fracture did happen and we had to remove the tumor surgically. Autograft of the fibula was done and then internally fixed.

PBL has a favorable prognosis over patients of systemic lymphoma with bone involvement. Age is a prognostic factor. The majority of the reports indicate better survival for patients younger than 60 years, but there are a few other reports that show better survival in patients younger than 40 or 50 years [4]. Jawad *et al*. analyzed 1,500 adult patients with PBL, and pointed out that younger age and localized disease were independent predictors of survival [14]. The study also noted that the incidence of the disease has increased during recent years [14]. Future treatment for patients with PBL needs to be dependent on strict staging criteria and adherence to successful published protocols using collaborative clinical trials.

## Conclusion

PBL is a rare but distinct clinicopathological entity. Compared with single treatment, CMT can result in both a good local control rate and survival benefits for local PBL.

## Consent

Written informed consent was obtained from the patient for publication of this case report and accompanying images. A copy of the written consent is available for review by the Editor-in-Chief of this journal.

## Abbreviations

CMT: Combined modality treatment; DFS: Disease-free survival; FDG: Fluorodeoxyglucose; PBL: Primary bone lymphoma; R-COP: Rituximab, cyclophosphamide, vincristine, prednisone; R-CHOP: Rituximab, cyclophosphamide, doxorubicin, vincristine, prednisone.

## Competing interests

The authors declare that they have no competing interests.

## Authors’ contributions

ML was a major contributor in writing the manuscript. BL critically revised the manuscript. FH reviewed the PBL treatment. YS reviewed the literature and gave final approval of the version to be submitted. All authors read and approved the final manuscript.

## Authors’ information

ML and BL are the joint first authors.
